# Vitrification of immature bovine cumulus-oocyte complexes: effects of cryoprotectants, the vitrification procedure and warming time on cleavage and embryo development

**DOI:** 10.1186/1477-7827-10-73

**Published:** 2012-09-06

**Authors:** Jennifer R Prentice-Biensch, Jaswant Singh, Reuben J Mapletoft, Muhammad Anzar

**Affiliations:** 1 Agriculture and Agri-Food Canada, Saskatoon Research Centre, Saskatoon, Saskatchewan, Canada; 2 Department of Veterinary Biomedical Sciences, Western College of Veterinary Medicine, University of Saskatchewan, Saskatchewan, Saskatoon, Canada; 3 Department of Large Animal Clinical Sciences, Western College of Veterinary Medicine, University of Saskatchewan, Saskatchewan, Saskatoon, Canada

**Keywords:** Cattle, Cumulus-oocyte-complexes, Germinal vesicle stage, Cryoprotectants, Ethylene glycol, Dimethyl sulfoxide, Vitrification, Warming time, *In vitro* maturation, *In vitro* fertilization

## Abstract

**Background:**

The present studies evaluated the effects of cryoprotectants, the vitrification procedure and time in the warming solution containing sucrose on cleavage and embryo development of immature (GV stage) bovine cumulus-oocyte complexes (COCs).

**Methods:**

Two experiments were conducted. In Experiment 1, COCs (n = 420) were randomly assigned to four groups: 1) Control group: no treatment; 2) VS1 group: COCs were exposed to vitrification solution 1 (VS1) containing 7.5% ethylene glycol [EG] + 7.5% dimethyl sulfoxide [DMSO] + 20% calf serum [CS] in TCM-199 at 37 C for 5 min; 3) VS1 + VS2 group: COCs were exposed to VS1 for 5 min followed by VS2 (15% EG + 15% DMSO + 17.1% sucrose + 20% CS) at 37 C for 45–60 sec; and 4) Vitrified group: COCs were exposed to VS1 and VS2, loaded on cryotops, vitrified in liquid nitrogen and then warmed in TCM-199 + 17.1% sucrose + 20% CS at 37 C for 1 min. In Experiment 2, COCs (n = 581) were assigned to the same groups, but those in VS1, VS1 + VS2 and Vitrified groups were sub-divided and exposed to the warming solution for either 1 or 5 min. After treatment and/or warming, all COCs in both experiments underwent *in vitro* maturation, *in vitro* fertilization and *in vitro* culture.

**Results:**

Cleavage and blastocyst rates did not differ among Control, VS1 and VS1 + VS2 groups in either experiment. In Experiment 2, there was no effect of time in the warming solution.

However, both cleavage and blastocyst rates were lower (*P* < 0.001) in the Vitrified group than in the Control, VS1 and VS1 + VS2 groups (40.9 and 1.6% vs 92.2 and 34.4%, 79.4 and 25.2%, and 80.2 and 20.8%, respectively in Experiment 1, and 25.0 and 1.7% vs 75.3 and 27.2%, 67.9 and 19.5%, and 62.7 and 22.5%, respectively in Experiment 2).

**Conclusions:**

The permeating cryoprotectants (EG and DMSO) present in VS1 and VS2 solutions and the time in the warming solution containing sucrose had no adverse effects on cleavage and blastocyst rates of immature bovine COCs. However, cleavage rate and early embryo development were reduced following the vitrification and warming.

## Background

Development of a reliable method for the cryopreservation of oocytes is important for assisted reproduction in human fertility clinics [1] and for conservation of female animal genetic resources [2-4]. Oocyte cryopreservation has been performed in several mammalian species; however, success rates have been low due to oocytes’ unique structure and sensitivity to cooling [5]. Slow freezing and vitrification are two common methods of cryopreservation for mammalian oocytes and embryos. In conventional, slow (controlled) freezing, cells dehydrate because of increased salt concentrations in the extracellular compartment resulting in a reduced likelihood of intracellular ice formation, but this results in increased intracellular salt concentrations referred to as the “solution effect” which can also cause cell damage [6]. Alternately, vitrification exposes cells to relatively high concentrations of cryoprotectants and ultra-rapid cooling [7]. Vitrification is used to avoid chilling injury and ice crystal formation in the cryopreservation of tissue, embryos and oocytes [8-10]. Although vitrification does not require a sophisticated and expensive programmable cell freezer, and is a fairly quick procedure, it requires skill and experience.

Vitrification involves ultra-rapid cooling and results in glass formation due to high viscosity of the cryoprotectants in intra- and extra-cellular compartments [11,12]. Although mouse oocytes have been vitrified successfully [13], vitrification of bovine oocytes is challenging due to their complex structure and sensitivity to chilling [14]. Previously, we examined the effect of vitrification of bovine oocytes on nuclear maturation, cleavage and blastocyst development [15] and found that vitrified oocytes had reduced embryo developmental competence, as indicated by low blastocyst production rates (<5%) compared to non-vitrified controls (31%).

One of the biophysical factors causing cellular disruption during cryopreservation is intracellular ice formation. However, vitrification reduces or eliminates intracellular ice formation by using highly viscous cryoprotectant solutions at relatively high concentrations [12,16]. Ethylene glycol, propylene glycol, glycerol and dimethyl sulfoxide [17] are used as permeating cryoprotectants for oocyte vitrification. Despite the critical importance of cryoprotectants for avoiding ice crystal formation in oocytes, the high concentration of cryoprotectants required for vitrification may be toxic and may cause osmotic injury to the cells, leading to reduced developmental competence [11,18]. To investigate the reduced developmental competence of oocytes following vitrification, it is important to examine the individual components of the vitrification system. Therefore, we proposed to determine the role of high concentrations of cryoprotectants present in vitrification solutions on cleavage rate of vitrified bovine oocytes and subsequent embryo development.

Warming time is a function of the temperature of the warming solution, and the sample volume and thickness which affects heat transfer. During the warming process, the oocytes are brought to the same temperature as the warming solution at an extremely high rate. The exposure to the warming solution is essential not only for warming, but also for removing cryoprotectants and rehydrating the oocytes [19]. Oocyte swelling during warming is an important determining factor for survival as volume fluctuations affect the plasma membrane integrity and organization of the cytoskeleton [20]. When exposed to an isotonic solution directly, water diffuses into the cell more rapidly than the cryoprotectants flow out and osmotic swelling can occur beyond the volume limits of the oocyte, resulting in membrane damage [21]. Thus, the warming solution normally contains a nonpermeating cryoprotectant such as sucrose [22,23]. If the time in the warming solution is insufficient, cryoprotectants may not be completely removed; however, the appropriate time interval for vitrified oocytes in the warming solution is still unclear. With this background, we hypothesized that 1) the high concentrations of cryoprotectants used in vitrification procedures would have toxic effects on bovine cumulus-oocyte complexes (COCs), resulting in low cleavage and embryonic developmental rates, and 2) exposing vitrified COCs to the warming solution containing sucrose for a longer interval would improve cleavage and blastocyst production rates by permitting the complete removal of intracellular cryoprotectants. Therefore, the present studies aimed to investigate the effects of the exposure of bovine COCs to vitrification solutions, the vitrification procedure, and time in the warming solution on cleavage and subsequent embryo development following *in vitro* fertilization.

## Methods

This study was approved by the Animal Care Committee and Animal Research Ethics Board, University of Saskatchewan, Saskatoon, Canada.

### Chemicals and supplies

Dulbecco’s phosphate buffered saline (DPBS), newborn calf serum (CS), TCM-199 and MEM non-essential amino acids were purchased from Invitrogen Inc. (Burlington, ON, Canada). Lutropin-V (LH) and Folltropin-V (FSH) were supplied by Bioniche Animal Health Inc. (Belleville, ON, Canada). Unless otherwise stated, all other chemicals and reagents were purchased from Sigma-Aldrich (Oakville, ON, Canada).

### Collection and initial processing of COCs

Bovine ovaries were collected from a slaughterhouse and transported to the laboratory at approximately 25°C within 8 h. Ovaries were washed in 0.15 M NaCl and extra-ovarian tissues were removed. The immature COCs at the germinal vesicle (GV) stage were aspirated from follicles (3–8 mm in diameter) using an 18-gauge needle attached to a 5 ml syringe containing approximately 1.0 ml of DPBS supplemented with 5% CS (v/v). The aspirated follicular fluid was pooled in 50 ml conical tubes and allowed to settle. The COCs were located under a stereomicroscope at 10x magnification, washed 3 times in DPBS + 5% CS and those with more than three layers of compacted cumulus cells and uniform cytoplasm (Grade 1) were selected for further processing.

### Vitrification and warming procedures

The COCs (GV stage) were vitrified by first equilibrating in vitrification solution 1 [VS1; TCM-199 + 7.5% ethylene glycol (EG; v/v) + 7.5% dimethyl sulfoxide (DMSO; v/v) + 20% CS] for 5 min at 37 C. After equilibration, COCs were transferred through three 20 μl-drops of vitrification solution 2 [VS2; TCM-199 + 15% EG (v/v) + 15% DMSO (v/v) + 20% CS (v/v) + 17.1% sucrose (w/v)] at 37 C for 45–60 sec [10,24]. Five COCs were loaded on each cryotop (Kitazato Supply Co., Fujinomiya, Japan) under a stereomicroscope with minimum surrounding medium and immediately plunged into liquid nitrogen [9]. The COCs were warmed by immersing the cryotop in 2 ml of the warming solution [TCM-199 + 20% CS (v/v) and 17.1% sucrose (w/v)] in a 35 mm petri dish at 37°C for 1 min. The COCs were then washed 3 times in DPBS supplemented with 5% CS (v/v) at 37°C.

### In vitro maturation, fertilization and culture

*In vitro* maturation, fertilization and culture procedures were conducted as previously described [15]. Briefly, the immature COCs (GV stage) were washed 3 times in maturation medium [TCM-199 supplemented with 5% CS, 5 μg/ml LH, 0.5 μg/ml FSH and 0.05 μg/ml gentamicin]. For *in vitro* maturation (*IVM*), groups of 20 COCs were placed in 100 μl droplets of maturation medium, under mineral oil and incubated for 22 h at 38.5°C, 5% CO_2_ in air and saturated humidity. For *in vitro* fertilization (*IVF*), semen from three fertile bulls (2 straws/bull) was thawed at 37 C for 1 min, pooled and washed through Percoll gradient (45% and 90%) [25]. After washing, spermatozoa were added to Brackett-Oliphant (BO) fertilization medium [26] to a final concentration 3 x 10^6^ sperm/ml. Following IVM, groups of 20 mature COCs were washed 3 times in BO supplemented with 10% bovine serum albumin (BSA, w/v) and added to 100 μl droplets of spermatozoa in BO, under mineral oil. A parthenogenetic control group of COCs (metaphase II stage) was incubated with no sperm. After 18 h of co-incubation with spermatozoa at 38.5°C, 5% CO_2_ in air and saturated humidity, cumulus cells and sperm attached to oocytes were mechanically removed via pipetting. The presumptive zygotes were washed 3 times through *in vitro* culture (*IVC*) medium consisting of CR1aa [5% CS, 2% BME essential amino acids (v/v), 1% MEM nonessential amino acids (v/v), 1% L-glutamic acid (v/v), 0.3% BSA and 0.05 μg/ml gentamicin sulfate]. Then, 20–25 zygotes were transferred into 100 μl IVC droplets under mineral oil and incubated at 38.5°C under 5% CO_2_, 90% N_2_, 5% O_2_ and saturated humidity. After 48 h in culture, the cleavage (2–8 cells) rate was recorded, and embryo culture was continued in the same droplets. Subsequently, the blastocyst (containing inner cell mass, trophoblast and blastocoel) rate was determined on Days 7, 8 and 9 (Day 0 = day of *IVF*). Both cleavage and blastocyst rates were based on the total number of COCs (GV-Grade 1) used in each treatment.

### Experiment 1: Effects of cryoprotectants and vitrification on cleavage and embryo development

The immature COCs (GV stage) were assigned randomly to the following four groups: 1) Control group - COCs were held in DPBS supplemented with 5% CS; 2) VS1 group - COCs were exposed to VS1 for 5 min and then placed in DPBS supplemented with 5% CS; 3) VS1 + VS2 group - COCs were exposed to VS1 for 5 min followed by VS2 for 45–60 sec, and then placed in DPBS supplemented with 5% CS; 4) Vitrified group - COCs were exposed to VS1 for 5 min, VS2 for 45–60 sec, vitrified in liquid nitrogen using the cryotop method, warmed at 37°C in the warming solution containing 17.1% sucrose (w/v) for 1 min and transferred in DPBS supplemented with 5% CS. After treatments, all COCs underwent *IVM, IVF* and *IVC* as described above. Cleavage and blastocyst rates were used as evaluation parameters. This experiment was replicated five times on different dates.

### Experiment 2: Effects of cryoprotectants, vitrification procedure, and time in the warming solution on cleavage and embryo development

As in Experiment 1, COCs were randomly assigned to Control, VS1, VS1 + VS2 and Vitrified groups; however, the COCs in VS1, VS1 + VS2 and Vitrified groups were sub-divided and exposed to the warming solution containing 17.1% sucrose (w/v) for either 1 or 5 min, followed by DPBS supplemented with 5% CS. All COCs then underwent *IVM, IVF and IVC*. Cleavage and blastocyst rates were used as evaluation parameters. This experiment was replicated five times on different dates.

### Statistical analysis

Data were analyzed using Proc Glimmix in SAS® Enterprise Guide 4.2 [27]. Analyses were performed using randomized complete block design modeling binary distribution (for yes/no response variables) for cleavage and blastocyst rates. The effects of Control, VS1, VS1 + VS2 and Vitrification were examined for cleavage and blastocyst rates in Experiment 1. In Experiment 2, analyses were performed using 4 x 2 factorial randomized complete block design. Replicate number (1 to 5), treatment (1 = control, 2 = VS1, 3 = VS1 + VS2, 4 = vitrified), warming time (1 = 1 min warming time, 2 = 5 min warming time) and binomial response (1 = cleavage or blastocyst, 2 = no cleavage or no blastocyst development) were recorded for each oocyte. Syntax of the SAS program was: Proc glimmix method = quad; class replicate treatment warming_time; model cleavage (event = "1") = treatment|warming_time / dist = bin link = logit; random intercept/ subject = replicate; run. If the P-value for treatment, warming_time or their interaction term from Type III sum of squares was < 0.05, then “lsmeans treatment warming_time treatment*warming_time/ diff lines ilink or adjust = tukey” was added to the syntax for separation of group means.

## Results

### Experiment 1: Effects of cryoprotectants and vitrification on cleavage and embryo development

Data on the effects of exposure to vitrification solutions and vitrification on oocyte cleavage and embryo development are presented in Table 1. The cleavage rate of oocytes in the Control (not exposed to cryoprotectants or cooling; 92.2%), VS1 (79.4%) and VS1 + VS2 (80.2%) groups did not differ, but all were higher than in the Vitrified group (40.9%; *P <* 0.001). Similarly, the blastocyst rate (% of total number of oocytes that were vitrified) in the Control (34.4%), VS1 (25.2%) and VS1 + VS2 (20.8%) groups did not differ, but all were higher (*P <* 0.001) than the Vitrified group (1.6%).

**Table 1:** Effects of cryoprotectant exposure and vitrification on cleavage of bovine oocytes and subsequent embryo development (Experiment 1)

**Group**	**GV oocytes (n)**	**Cleavage rate n (%)**	**Blastocyst rate n (% of total)**
*Control (non-exposed)*	90	83 (92.2%)^a^	31 (34.4%)^a^
*VS1*	107	85 (79.4%)^a^	27 (25.2%)^a^
*VS1 + VS2*	96	77 (80.2%)^a^	20 (20.8%)^a^
*Vitrified*	127	52 (40.9%)^b^	2 (1.6%)^b^
*P-value*			
Cryoprotectant exposure		0.847	0.456

Values with different superscripts (a,b) in the same column differ (*P <* 0.05).

VS1: Vitrification solution 1.

VS2: Vitrification solution 2.

### Experiment 2: Effects of cryoprotectants, vitrification procedure, and time in the warming solution on cleavage and embryo development

Data on the effects of cryoprotectant exposure, vitrification and time in the warming solution on cleavage and blastocyst rates are presented in Table 2. Within treatment group, there was no effect of time in the warming solution on cleavage or subsequent embryo development. When data from the Vitrified group were analyzed separately for the effect of time in the warming solution (after excluding all other groups), there was also no effect on cleavage rate or blastocyst rate. Both cleavage and blastocyst rates (% of total number of oocytes that were vitrified) in the Vitrified group (25.0 and 1.7%, respectively) were lower (*P <* 0.001) than in non-vitrified Control (75.3 and 27.2%, respectively), VS1 (67.9 and 19.5%, respectively) or VS1 + VS2 (62.7 and 22.5%, respectively) groups which did not differ from one another.

**Table 2:** Effects of cryoprotectant exposure, vitrification and time in the warming solution on cleavage of bovine oocytes and subsequent embryo development (Experiment 2)

**Group**	**GV oocytes (n)**	**Cleavage rate n (%)**	**Blastocyst rate n (% of total)**
*Control (non-exposed)*	81	61 (75.3%)^a^	22 (27.2%)^a^
*VS1*			
* 1 min warming interval*	82	60 (73.2%)^a^	16 (19.5%)^a^
* 5 min warming interval*	77	48 (62.3%)^a^	15 (19.5%)^a^
*VS1 + VS2*			
* 1 min warming interval*	87	52 (59.8%)^a^	16 (18.4%)^a^
* 5 min warming interval*	82	54 (65.9%)^a^	22 (26.8%)^a^
*Vitrified*			
* 1 min warming interval*	74	22 (29.7%)^b^	1 (1.4%)^b^
* 5 min warming interval*	98	21 (21.4%)^b^	2 (2.0%)^b^
*P-value*			
Cryoprotectant exposure		0.202	0.205
Warming time		0.241	0.689
Cryoprotectant exposure * warming time		0.381	0.687

Values with different superscripts (a,b) in the same column differ (*P <* 0.05).

VS1: Vitrification solution 1.

VS2: Vitrification solution 2.

No cleavage or blastocyst development was observed in the parthenogenetic control group in either Experiment 1 or 2.

## Discussion

In order to achieve intracellular vitrification, there are three main requirements i.e., high concentrations of permeating cryoprotectants, ultra-rapid cooling and low sample volume. Alteration in any one of these components can affect the success of the vitrification process. High concentrations of cryoprotectants are used to increase the viscosity of intra- and extra-cellular solutions, which increases the glass transition temperature [28]. Ethylene glycol (EG) and DMSO are commonly used cryoprotectants in vitrification, but they are considered toxic due to their cell permeating nature and the high concentrations needed to induce vitrification [29]. Ultra-rapid cooling allows the addition of permeating cryoprotectants at lower concentrations reducing toxicity to the cells [30]. Decreasing the sample volume during the vitrification procedure increases the cooling rate which improves heat transfer [29].

In the present study, the most profound and consistent observation was that the high concentrations of cryoprotectants used (15% EG and DMSO) had no adverse effect on cleavage and blastocyst production rates. Similarly, exposure of vitrified oocytes to the warming solution containing sucrose for 5 min did not affect cleavage and blastocyst production, as compared to 1 min, regardless of group. On the contrary, vitrification of the GV stage oocytes affected cleavage rates and blastocyst rates profoundly following in vitro maturation and fertilization. In both experiments, the cleavage rates were significantly reduced in the Vitrified group and blastocyst rates were not only significantly reduced, but were exceptionally low. Although these experiments were not designed to determine the necessity of sucrose in the warming solution, it is difficult to imagine that the absence of sucrose in the warming solution could be anything but detrimental. Thus, we concluded that our vitrification procedure was deficient in one or more ways for the cryopreservation of germinal vesicle stage bovine oocytes.

In the current studies, cleavage and blastocyst production rates in the VS1 + VS2 group did not differ from that in the Control group (Table 1). This suggests that the 45–60 sec exposure time was not sufficient for permeating these cryoprotectants to penetrate inside the bovine oocytes [31], or if penetrated, DMSO and EG had no toxic effects on bovine oocytes. Although there is still considerable debate, published literature tends to confirm the likelihood that these cryoprotectants are not highly toxic. DMSO alone [32] or in combination with other cryoprotectants [33] has been used successfully for oocyte vitrification, and in at least one study [34], the exclusion of DMSO from vitrification solution resulted in lower survival rates of oocytes. However, it has also been reported that DMSO (used alone or in combination with EG) resulted in reduced developmental competence of oocytes [11,18]. In other studies, DMSO-free vitrification systems e.g., glycerol and propanediol [35,36], or ethylene glycol and sucrose [37-39], have provided promising results with oocytes [34].

An ultra-rapid cooling rate is generally considered as one of the requirements for successful vitrification. Recently, ultra-rapid warming has been reported to be more important than concentrations of cryoprotectants or cooling rate [40]. The optimal warming time for vitrified bovine oocytes is still unknown. However, exposing oocytes to the warming solution for 5 min as opposed to 1 min in Experiment 2 appeared to have no obvious benefit on subsequent embryo development. Therefore, 1 min appears to be a sufficient time in the warming solution. It is also noteworthy that exposure to the warming solution containing sucrose for either 1 or 5 min did not have any adverse effect in nonvitrified oocytes in the VS1 and VS1 + VS2 groups.

Osmotic stress resulting from exposure to high concentrations of cryoprotectants in vitrification solutions could cause oocytes to undergo dramatic volume changes during warming. In this regard, one of the most important factors affecting cell survival during dilution is excessive cell swelling. Water moves through the cell membrane more rapidly than cryoprotectants, and because of the high concentration of cryoprotectants remaining in the cell, swelling occurs. Oocytes have biological limits in their tolerance to the osmotic stress associated with high concentrations of cryoprotectants [23]. Volume fluctuations can affect the integrity of the plasma membrane as well as the cytoskeletal organization of oocytes [20]. During this process, oocytes are rehydrated while cryoprotectants and water are exchanged due to their gradients across the cell membrane [19]. For this reason, sucrose is commonly used in warming solution to counterbalance the osmotic shock during conventional freezing [22] and vitrification [23]. Although these experiments were not designed to determine the benefits of sucrose in the warming solution, there was no evidence for any adverse effects.

Bovine oocytes at different nuclear stages (germinal vesicle, metaphase I and metaphase II) have been cryopreserved successfully, using both slow freezing or vitrification methods. Mature oocytes have meiotic spindles that are extremely sensitive to cryoprotectant additives and cooling rates which result in tubulin depolymerization [11,41,42]. Therefore, immature oocytes (GV stage) enclosed in cumulus cells (COCs) were used in the current study. The role of cumulus cells during oocyte cryopreservation is still not clear. Cumulus cells provide a rigid structure to oocytes and thus protect them against osmotic shock during cryopreservation [43]. It has been reported that the survival, cleavage and blastocyst production rates of bovine oocytes were higher when vitrified with enclosed cumulus cells than partially denuded COCs [10], as cumulus cells support the oocyte during *in vitro* maturation and fertilization [44].

Cryopreservation of oocytes has been reported to result in several ultrastructural and morphological alterations including damage to the cell membrane and ooplasm, and abnormal distribution of chromosomes, microtubules and actin microfilaments [45-47]. Additionally, damage to gap junction integrity can result in disruption of the communication between cumulus cells and oocytes [11,48]. These morphological changes have been linked to a failure in fertilization and embryo development [48,49]. Consequently, the disruptions in protein synthesis, and the failure of cumulus cell expansion and further meiotic development may also be responsible for the failure of cleavage and subsequent embryo development following vitrification [50].

In a recent study, only 23% of vitrified COCs (GV stage) reached the MII stage following IVM as compared to 84% in nonvitrified control COCs [51]. In another study using similar vitrification conditions (15% EG and DMSO in VS2, 45–60 sec exposure time and cryotop as a loading device), cleavage and blastocyst production rates were somewhat higher than in the present study, but still low [10]. It is noteworthy that in the present study, cleavage and blastocyst rates were based on the original number of oocytes (COCs) that were vitrified. Thus, data have been presented and interpreted very conservatively. Based on these results and published literature, we must conclude that the optimum vitrification procedure for GV stage oocytes has yet not been developed.

## Conclusions

The brief exposure (45–60 sec) of immature (GV stage) bovine COCs to vitrification solutions (15% of each EG and DMSO) did not adversely affect their subsequent cleavage and embryo development. There was no apparent cytotoxic effect and there was no evidence that osmotic stress was responsible for the low blastocyst production rates following vitrification. Furthermore, 5 min exposure to the warming solution had no added benefits over 1 min, with regards to cleavage and blastocyst production rates. Collectively, data provide evidence suggesting that the vitrification procedure was responsible for the reduced cleavage and embryo development rates following vitrification and warming.

## Abbreviations

COCs: Cumulus-oocyte complexes; GV: Germinal vesicle; VS: Vitrification solution; EG: Ethylene glycol; DMSO: Dimethyl sulfoxide; CS: Calf serum; TCM-199: Tissue Culture Medium-199; IIF: Intracellular ice formation; DPBS: Dulbecco’s phosphate buffered saline; MEM: Minimum Essential Medium; IVM: In vitro maturation; IVF: In vitro fertilization; BO: Brackett-Oliphant; IVC: In vitro culture; BSA: Bovine serum albumin.

## Competing interests

The authors declare that they have no competing interests.

## Authors’ contributions

JRP conducted the experiments, analyzed the data and wrote the draft of manuscript. JS designed the experiment, and analyzed the data and interpreted the results. RJM designed the experiment and revised the manuscript critically. MA designed and coordinated the whole study, interpreted the data, prepared the manuscript, acted as an MSc supervisor of JRP. All authors read and approved the final manuscript.
